# Persistency of Mesenchymal Stromal/Stem Cells in Lungs

**DOI:** 10.3389/fcell.2021.709225

**Published:** 2021-07-16

**Authors:** Erica Ferrini, Fabio Franco Stellari, Valentina Franceschi, Francesca Macchi, Luca Russo, Alba Murgia, Giulia Grisendi, Gino Villetti, Massimo Dominici, Gaetano Donofrio

**Affiliations:** ^1^Chiesi Farmaceutici S.p.A., Corporate Pre-Clinical R&D, Parma, Italy; ^2^Department of Veterinary Science, University of Parma, Parma, Italy; ^3^Department of Medical and Surgical Sciences for Children and Adults, University of Modena and Reggio Emilia, Hospital of Modena, Modena, Italy; ^4^Scientific and Technological Park of Medicine “Mario Veronesi,” Mirandola, Italy

**Keywords:** MSCs, BM-MSCs, lung, MSCs tracking, BLI

## Abstract

Mesenchymal stromal/stem cells (MSCs) are a fibroblast-like cell population with high regenerative potential that can be isolated from many different tissues. Several data suggest MSCs as a therapeutic tool capable of migrating to a site of injury and guide tissue regeneration mainly through their secretome. Pulmonary first-pass effect occurs during intravenous administration of MSCs, where 50 to 80% of the cells tend to localize in the lungs. This phenomenon has been exploited to study MSC potential therapeutic effects in several preclinical models of lung diseases. Data demonstrated that, regardless of the lung disease severity and the delivery route, MSCs were not able to survive longer than 24 h in the respiratory tract but still surprisingly determined a therapeutic effect. In this work, two different mouse bone marrow-derived mesenchymal stromal/stem cell (mBM-MSC) lines, stably transduced with a third-generation lentiviral vector expressing luciferase and green fluorescent protein reporter genes tracking MSCs *in vivo* biodistribution and persistency, have been generated. Cells within the engrafted lung were *in vivo* traced using the high-throughput bioluminescence imaging (BLI) technique, with no invasiveness on animal, minimizing biological variations and costs. *In vivo* BLI analysis allowed the detection and monitoring of the mBM-MSC clones up to 28 days after implantation independently from the delivery route. This longer persistency than previously observed (24 h) could have a strong impact in terms of pharmacokinetics and pharmacodynamics of MSCs as a therapeutic tool.

## Introduction

At the beginning of their discovery, 1970s, mesenchymal stromal/stem cells (MSCs) were described as a bone marrow population of cells with fibroblastic and clonogenic properties; for these reasons, they were defined as “colony-forming unit fibroblasts” (Friedenstein et al., 1970, 1974, 1976). Currently, MSCs can be obtained from many tissues, and minimal criteria for their definition were internationally established (Dominici et al., 2006). They should have: (1) fibroblastic-like morphology; (2) adhesion properties on plastic; (3) the capacity to differentiate down to chondrogenic, osteogenic, and adipogenic lineages; and (4) the ability to express CD105, CD73, CD44, and CD90 and not express CD11b, CD79α/CD19, and HLA-DR surface markers (Dominici et al., 2006).

Mesenchymal stromal/stem cells have been shown to exert therapeutic potential through their secretome (comprehensive complex array of components ranging from soluble secreted factors to factors encapsulated in extracellular vesicles with anti-inflammatory as well as growth properties) and through the capability of migrating to the site of tissue injury, a characteristic that involves a contact-dependent mechanism of action (Harrell et al., 2019a,b). Recently, it has been observed in a sepsis model that heat-inactivated and secretome-deficient MSCs were still capable of inducing monocyte recognition, phagocytosis, and selective apoptosis. Interestingly, these activities were passively enabled by the MSC structures (Weiss et al., 2020). MSCs exhibit a diverse array of effects and multifunctional mechanisms of action, with no single and overarching biological effect. Probably, a complicated network, orchestrated by MSCs and in relation to nearby cells, of the injured tissue generates a biological reaction out-coming in a “therapeutic effect.” Many strategies have been adopted to potentiate MSC therapeutic profile and reduce the amount of cells administered with the aim of reducing procedure costs and increase their safety for the patient. These strategies include optimizing culture conditions, MSC activation before administration, and, probably the most important, alteration of MSC biodistribution.

When MSCs are intravenously administered, most of them (∼50–80%) tend to localize to the lung (first-pass effect; Barbash et al., 2003; Kraitchman et al., 2005; Fischer et al., 2009; Assis et al., 2010) due to their large size (∼20–30 μm of diameter; Gao et al., 2001; Sekiya et al., 2002; Fischer et al., 2009), larger than circulating immune cells and lung capillary (Crop et al., 2010). Furthermore, the number of trapped cells decreased with the administration of a vasodilator (Gao et al., 2001; Sekiya et al., 2002; Fischer et al., 2009), supporting the hypothesis that MSC size is a major contributor to the first-pass effect.

This lung trapping, although considered an obstacle when MSCs need to be delivered to organs other than the lung, was exploited for repair, remodeling, and/or regenerating damaged lungs. Preclinical therapeutic models exploiting MSCs for different lung diseases, such as idiopathic pulmonary fibrosis (IPF; Tanaka et al., 2014), bronchopulmonary dysplasia (Aslam et al., 2009), cystic fibrosis (Loi et al., 2006), chronic lower respiratory disease (Feizpour et al., 2014), and acute respiratory distress syndrome (Asmussen et al., 2014), have been generated. For each of the models accounted, regardless of the route of delivery [intravenous (IV), intratracheal (IT), intraperitoneal (IP), and intranasal], cells reaching the lung did not survive longer than 24 h (Eggenhofer et al., 2012; Horie et al., 2018), after which cells were cleared and, although in some cases limited, a therapeutic effect was surprisingly and still achieved. Based on these data, in the present work, MSC persistency in the lung was revised and a longer persistency was observed.

## Results

### Stable Labeling for *in vivo* Tracking of Mouse Mesenchymal Stromal/Stem Cells

**FIGURE 1:**
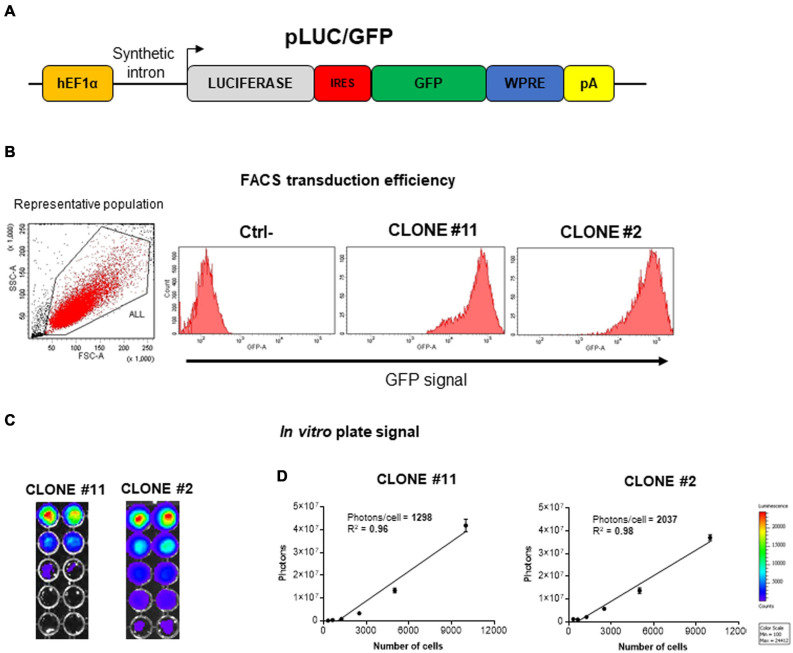
Generation and *in vitro* characterization of stably transduced mouse bone marrow-derived mesenchymal stromal/stem cell (mBM-MSC) clones. **(A)** Schematic representation not to scale of the pLuc/GFP third-generation lentiviral construct carrying an expression cassette containing luciferase (Luc, colored in gray) and enhanced green fluorescent protein (EGFP, colored in green) open reading frames (ORFs) linked by an internal ribosome entry site (IRES, colored in red) sequence and transcriptionally regulated by human Elongation Factor 1 alpha (hEF1α) promoter, along with a synthetic intron. **(B)** Assessment of GFP expression by fluorescent activated cell sorting (FACS) analysis of clone #2 and #11Luc/GFP lentivirus-transduced mBM-MSCs. Non-transduced cells are used as negative control (Ctrl-). **(C)** *In vitro* titration of bioluminescence emission from clone #2 and #11 by *In Vivo* Imaging System (IVIS) bioluminescence imaging system. Clone #2 emits higher proton titers compared to clone #11. **(D)** Plot of linear correlation between total photon emission (in *y* axis) and cell numbers (in *x* axis). Quantification of photons emitted per single cell allowed the identification of clone #2 as the brighter clone.

Before attempting mouse MSC (mMSC) lung localization *in vivo*, two different clones (#2 and #11) of C57BL/6 mouse bone marrow-derived mesenchymal stromal/stem cells (mBM-MSCs) were stably transduced/transgenized with two different reporter genes to easily track their biodistribution *in vivo*. A third-generation lentiviral vector delivering luciferase and green fluorescent protein (GFP) in a bicistronic transfer vector, pWPI (Addgene; https://www.addgene.org/), was generated by subcloning the luciferase open reading frame (ORF) upstream of an internal ribosomal entry site (IRES) followed by the GFP ORF and woodchuck hepatitis virus posttranscriptional regulatory element (WPRE; Figure 1A). VSVg pseudotyped lentiviral vector particles were reconstituted in HEK293T cells and mBM-MSC transduced with efficiency close to 100% of GFP expression, measured by flow cytometry analysis (Figure 1B). Furthermore, both clones nicely express luciferase, as monitored by *In Vivo* Imaging System (IVIS), with clone #2 (#2Luc/GFP) displaying a stronger signal intensity (2,037 photons/cell) with respect to clone #11 (#11Luc/GFP; 1,298 photons/cell; Figures 1C,D). However, this difference could not be attributed to general biological differences between the two clones but probably only limited to transgene expression due to a different site of integration of the provirus genome into the host genome or different provirus genome copy number integrated. Although this is an interesting issue, but because it is out of the purpose of this work, it was not further investigated.

### Stable Transduction of Mouse Mesenchymal Stromal/Stem Cells Allows *in vivo* Direct Monitoring of Mouse Mesenchymal Stromal/Stem Cell Lung Persistency by Bioluminescence Imaging

**FIGURE 2:**
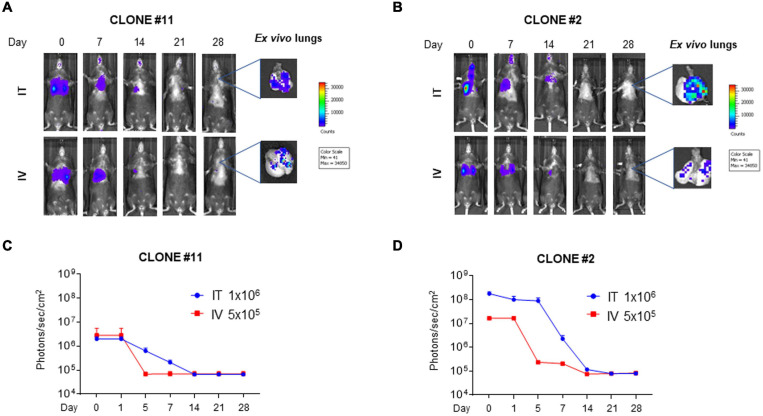
*In vivo* and *ex vivo* imaging of mouse bone marrow-derived mesenchymal stromal/stem cells (mBM-MSCs) in mouse lung. **(A,B)** *In vivo* bioluminescence imaging (BLI) of four groups of mice (*n* = 5 each) intratracheally (IT) and intravenously (IV) inoculated with mBM-MSCs clone #2 and #11 (**B,A**, respectively). Mice were weekly monitored up to 4 weeks post inoculation. At each time point, mice were intraperitoneally (IP) injected with 200 μl/mouse luciferin substrate to allow longitudinal detection of *in vivo* BLI. On day 28, mice were anesthetized and lungs were harvested for *ex vivo* imaging using *In Vivo* Imaging System (IVIS) BLI system. **(C,D)** BLI signal from mouse chest of each group was quantified seven times, expressed as photons/s/cm^2^ and plotted as mean ± standard error of the mean (SEM) using the software Living Image^®^ version 4.3.1. BLI signal at each time point represents the mean ± standard deviation of five animals. No significant differences were detected between IT and IV groups for either clone #11 or clone #2 (*p* > 0.05, two-way ANOVA test followed by Sidak’s test for multiple comparisons).

Although the clone #2Luc/GFP displayed a better luciferase expression with respect to the clone #11Luc/GFP, both clones were used for lung *in vivo* engraftment and to investigate if clonal selection could impact cell engraftment. #11Luc/GFP and #2Luc/GFP cells were transplanted *via* IV (5 × 10^5^ cells) or IT (10^6^ cells) in four different groups (*n* = 5) of C57BL/6 female mice and monitored by IVIS at different times (0, 1, 5, 7, 14, 21, 28 days) post implantation. In all groups of mice, independently from the route of administration and the cellular clone employed, cell localization into the lung was successful. #2Luc/GFP IT implanted cells were better detected in the lung (up to 7 days) with respect to #11Luc/GFP IT cells or #2Luc/GFP IV cells (Figures 2A,B) and well visualized, at least *ex vivo*, up to 28 days post implantation (Figures 2C,D).

### Transduced Mouse Bone Marrow-Derived Mesenchymal Stromal/Stem Cell Preadaptation Into the Lung Does Not Enhance Tissue Persistency

**FIGURE 3:**
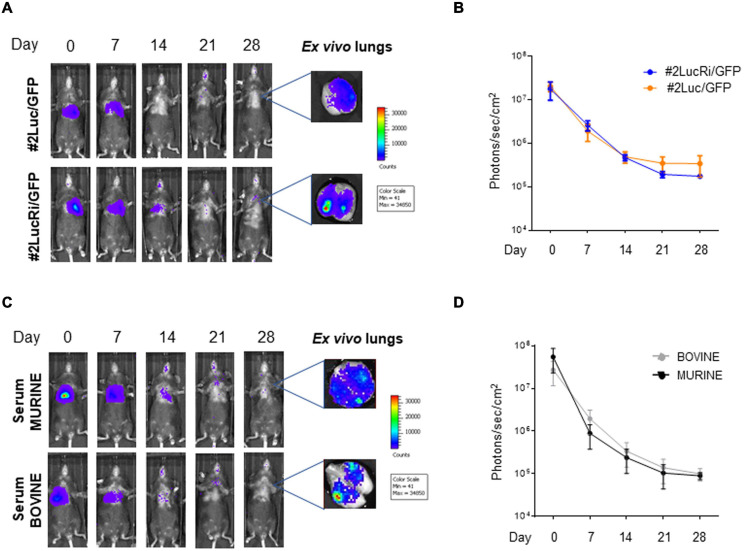
*In vivo* and *ex vivo* imaging of intratracheally (IT) injected mouse bone marrow-derived mesenchymal stromal/stem cells (mBM-MSCs). **(A)** *In vivo* bioluminescence imaging (BLI) of four groups of mice (*n* = 5 each) IT inoculated with mBM-MSC clone #2Luc/GFP or clone #2LucRi/GFP. Mice were weekly monitored up to 4 weeks post inoculation. At each time point, mice were longitudinally detected for *in vivo* BLI. On day 28, mice were anesthetized and lungs were harvested for *ex vivo* imaging using *In Vivo* Imaging System (IVIS) BLI system. No significant differences in survival were detected between mice transplanted with clone #2LucRi/GFP and mice transplanted with parental clone #2Luc/GFP (*p* > 0.05, two-way ANOVA test followed by Sidak’s test for multiple comparisons). **(B)** BLI signal from mouse chest of each group was quantified six times, expressed as photons/s/cm^2^ and plotted as mean ± standard error of the mean (SEM) using the software Living Image^®^ version 4.3.1. BLI signal at each time point represents the mean ± standard deviation of five animals. **(C)** *In vivo* longitudinal BLI quantification comparison between IT inoculated mice with clone #2LucRi/GFP differentially cultured with murine or bovine serum. **(D)** BLI signal from mouse chest of each group was quantified five times, expressed as photons/s/cm^2^, and plotted as mean ± SEM using the software Living Image^®^ version 4.3.1. BLI signal at each time point represents the mean ± standard deviation of five animals. No significant differences were detected between the two clones cultured in murine or bovine serum (*p* > 0.05, two-way ANOVA test followed by Sidak’s test for multiple comparisons).

Since #2Luc/GFP IT lung implanted cells could be better detected, with a decrease of signal intensity through time for up to 28 days, it was of interest to investigate whether preadaptation of the cells in the lung could select a cellular phenotype, due to biochemical stimuli received from the environment, able to survive longer when reimplanted. Fourteen days post #2Luc/GFP IT lung implantation, cells were isolated by lung tissue dissociation and green cells were sorted by fluorescent activated cell sorting (FACS). These cells, defined as #2LucRi/GFP, were first *in vitro* expanded and then characterized in terms of MSC markers/purity, proliferation potential, and differentiative capabilities/mesenchymal characteristics, compared to the parental clone #2Luc/GFP (data not shown). #2LucRi/GFP cells maintained MSC characteristics/purity and stable lentiviral transduction (Supplementary Figures 1A–C) besides a proliferating activity identical to #2Luc/GFP (Supplementary Figure 1D). Furthermore, when #2LucRi/GFP cells were transplanted IT into the mouse lung, they survived for the same period of time as the parental #2Luc/GFP cells (Figures 3A,B). Therefore, preadaptation of #2Luc/GFP into the lung did not impact their survival.

### Homologous Serum Does Not Impact Cell Persistence Into the Lung

Mouse bone marrow-derived mesenchymal stromal/stem cells are grown in the presence of fetal bovine serum (FBS), and cells tend to unspecifically endocytose serum proteins. These bovine proteins, hence antigens, could be released and activate a strong immune response when implanted into the mouse lung, thus compromising cell survival. #2Luc/GFP cells were incubated with medium containing murine homologous serum for 24 h in order to eliminate heterologous traces and then IT lung implanted. As shown in Figures 3C,D, although slight differences could be visually observed, no statistically significant differences in terms of engraftment were observed between #2Luc/GFP cells incubated with homologous serum with respect to those incubated with FBS. This was confirmed also between #2Luc/GFP and #2LucRi/GFP (data not shown).

### Host Genetic Background Does Not Impact Cell Persistency Into the Lung

**FIGURE 4:**
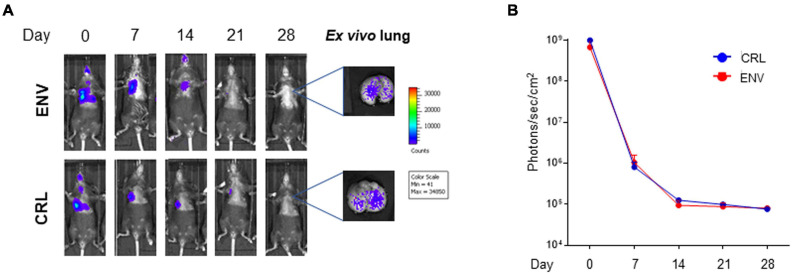
*In vivo* and *ex vivo* imaging of intratracheally (IT) injected mouse bone marrow-derived mesenchymal stromal/stem cells (mBM-MSCs) from different sources. **(A)** *In vivo* bioluminescence imaging (BLI) of IT mBM-MSC clone #2LucRi/GFP inoculated Charles River Laboratories (CRL) and Envigo (ENV) mice. Mice were weekly monitored up to 4 weeks post inoculation. On day 28, mice were anesthetized and lungs were harvested for *ex vivo* imaging using *In Vivo* Imaging System (IVIS) BLI system. No significant differences were detected between the two strains of mice, thus host genetic background does not impact cell persistency into the lung (*p* > 0.05, two-way ANOVA test followed by Sidak’s test for multiple comparisons). **(B)** BLI signal from each mouse was quantified five times, expressed as photons/s/cm^2^ and plotted as mean ± SEM. BLI signal at each time point represents the mean ± standard deviation of five animals.

Since the C57BL/6 host genetic background was syngenic with that of the transplanted cells, it was wondered if host mice, derived from different producers [Charles River Laboratories (CRL) and Envigo (ENV)], could create a difference in terms of MSC persistency. This might be due to very small genetic differences such as gene copy-number variants or single-nucleotide polymorphisms (SNPs). #2Luc/GFP cells were transplanted IT into two groups of mouse lung (CRL = 5 and ENV = 5) and, as observed in Figure 4, no differences in terms of persistency were observed between the two groups.

## Discussion

In the present work, mBM-MSC localization and persistency in the mouse lung were examined. mBM-MSCs were labeled *via* stable transduction with a replicating incompetent lentiviral vector delivering an expression cassette for luciferase and GFP. Since the luciferase ORF was in bicistronic form with GFP by an IRES sequence (Pelletier and Sonenberg, 1988; Hellen and Sarnow, 2001), the level of GFP expression in the transduced cells should reflect the expression level of the ORF upstream to the IRES, which, in this specific case, was luciferase. However, in most of the cases, the luciferase expression level is even higher with respect to that of the downstream GFP ORF (Pelletier and Sonenberg, 1988; Hellen and Sarnow, 2001). Using this strategy, mBM-MSCs expressing the highest amount of luciferase could be selected by simply sorting cells with the highest GFP expression, thus increasing the sensitivity of the system since bioluminescence imaging (BLI) was employed for detection.

Bioluminescence imaging is a technique based on the photo-detection of light generated by the enzymatic reaction of luciferase (expressed *in vivo* as a molecular reporter)-mediated oxidation of a molecular substrate. Photons emitted from the enzymatic reaction can be visualized as deep as several millimeters within tissues, allowing organ/tissue-level resolution. BLI is increasingly used by researchers for the *in vivo* visualization of different cellular events in the context of the whole animal body. This is due to its potency, reliability, low cost, high throughput, and relatively simple applicability. Inter-animal variation and errors are extremely reduced because a single animal can be repeatedly monitored with high resolution and less data wasting. Furthermore, the constant development of computational systems associated with BLI simplifies its application and can be used even by investigators with a restricted background in molecular imaging (Close et al., 2011; Franceschi et al., 2014). Taking advantage of an integrated bioluminescent overexpressed reporter gene into the genome of transduced mBM-MSCs and of BLI, we have been able to localize mBM-MSCs in the lung in real time up to 28 days, independently from the route of administration and from the amount of cells administered. There are two main methods to introduce cells into the lung: systemic delivery, IV, and local delivery, IT. Although IV represents the simplest delivery way, it raises a thromboembolic potential risk directly correlated to the number and the concentration of cells injected. In particular, concentration above 10^7^ cells/ml was shown, at least in mice, to generate a significant increase of pulmonary embolism and consequent mortality (Kyriakou et al., 2008). In our case, a single dose of 10^5^/200 μl cells was chosen because after several attempts, this amount of cells was shown to be the best compromise between risk of embolism and signal detection. Alternatively to the IV route, IT route was employed too. The lung is the only corporeal district where compounds can be directly delivered through the airways. Although previous works reported positive (Gupta et al., 2007; Wong et al., 2007, 2009) and negative (Kuang et al., 2005; Gupta et al., 2007) results, cell survival levels in these studies were performed using fluorescent techniques, which could justify these strong discrepancies. In our study, to determine if the airway might be a more effective route of mMSC delivery, 10^5^/50 μl cells were administered. Although the number of IT delivered cells was larger with respect to the IV ones, the cell persistency and signal kinetic we obtained in the lung were similar, even if a fraction of IT cells is generally coughed out by mucociliary clearance.

Even if with very low efficiency, it was shown that mMSCs derived from cytokeratin-18 (K18) promoter-driven GFP (K18GFP) transgenic mice, when delivered to the lung of wild-type recipient, could engraft and transdifferentiate in epithelial committed cells showing K18GFP transgene *in vivo* upregulation but not *in vitro* (MacPherson et al., 2006; Wong et al., 2007). These data suggest that the lung environment is responsible for lineage commitment and perhaps for long-term survival of mMSCs (MacPherson et al., 2006; Wong et al., 2007). Based on this assumption, mBM-MSCs were preadapted in the lung, re-isolated, and IT redelivered to the lung. Even though cell survival or at least their detectability did not vary, cells still persisted for 28 days.

A generally relevant aspect following MSC allogeneic or xenogenic transplantation is the recipient immune response against the transplanted cells. T cell and antibody production has been shown not only against the cells but also against FBS-derived antigens present in the cell media used to culture the cells (Leibacher and Henschler, 2016; Leibacher et al., 2017). We did substitute FBS in cell culture medium with mouse autologous serum 24 h before cell delivery into the lung. However, no substantial differences in terms of cell survival were observed.

C57BL/6 mouse genetic background is largely used for adoptive transfer experiments, and a large number of sublines with genetic polymorphisms are used around the world (Zurita et al., 2011), showing defined immune-phenotypic differences (Mahajan et al., 2016). Although it is difficult to define the impact that a genetic drift of a mouse-specific subline could have on mMSC survival when delivered to the lung, we limited our investigation to two sublines coming from two different mouse breeders, without observing significant differences.

Over the last 10 years, MSC therapy approach for lung diseases constantly progressed probably slower than expected with respect to the starting enthusiasm. Initially, a certain number of works, accompanied by a small body of noteworthy literature, suggested that exogenous MSCs, as well as other types of cells with a residual pluripotency, could engraft and differentiate into airway, alveolar epithelial, pulmonary vascular, and/or pulmonary interstitial cells (Gupta et al., 2007; Wong et al., 2007, 2009). It was hoped that when MSCs are delivered and engraftment takes place, they will differentiate, mature, and integrate into the target organs, inducing/resulting in tissue regeneration. Thanks to sophisticated technologies, researchers have been able to demonstrate that although MSCs can be induced *in vitro* to partially differentiate and express specific markers of alveolar or epithelial cells, this was a very rare or absent event *in vivo* (Pittenger et al., 2019). Since the initial idea of engraftment and differentiation has been largely abandoned and substituted with MSC immunomodulatory and paracrine actions, basic knowledge such as time of persistency of MSCs into the lung following systemic or local administration is still debated. This issue has strong implications in terms of pharmacokinetics and pharmacodynamics for MSCs with relevant therapeutic impact on their regenerative potential (Brooks et al., 2018; Aijaz et al., 2019; Salvadori et al., 2019). The data presented in this paper corroborate the MSC activity through their secretome in relation to their lung persistency. In an exciting paper recently published (Xie et al., 2021), the fundamental supporting signal of lung mesenchymal cells secretome for type 2 alveolar epithelial cell renewal in IPF-affected lung was demonstrated. Such supportive signal was further associated with growth hormone receptor expression and release *via* exosomes by lung mesenchymal cells. This observation, along with the longer persistency of exogenous MSCs in the lung we observed, could be exploited as cargo system for paracrine factors.

## Materials and Methods

### Cell Cultures

Mouse bone marrow-derived mesenchymal stromal/stem cells clones were isolated from C57BL/6 mice. Whole bone marrow cells were cultured at a density of 1 × 10^6^/cm^2^, and the plastic adherent fraction was defined as MSCs. These mBM-MSCs were characterized by the expression of typical MSC markers as CD29, CD49e, CD90, CD105, and Sca-1, whereas they lacked the expression of CD45. Cells were grown in complete Eagle’s Minimal Essential Medium (EMEM, Euroclone) containing 1 mM of sodium pyruvate (Gibco), 2 mM of L-glutamine (Sigma), 100 IU/ml of penicillin (Sigma), 100 μg/ml of streptomycin (Sigma), and 2.5 μg/ml of amphotericin B (Sigma), supplemented with 10% FBS and incubated at 37°C, 5% CO_2_. Every 3–4 days, mMSCs were passaged with 0.05% Trypsin-EDTA (Gibco) when they reached 80% confluence. In this study, we used mMSCs between passages 5 and 10.

### Lentiviral Construct Preparation and Lentivirus Reconstitution

The lentiviral construct pLuc/GFP was generated by subcloning the luciferase ORF, excised from the commercial vector pGL3basic (Promega) through *Hin*dIII/*Xba*I digestion and after a T4 polymerase blunting treatment, in the lentiviral third-generation pWPI vector (Trono Lab), opened with *Pme*I. pWPI is a bicistronic transfer vector able to efficiently deliver heterologous ORFs, in this case the luciferase ORF, upstream of an IRES sequence, followed by the GFP ORF and a WPRE.

Luc/GFP lentivirus has been reconstituted on HEK 293T cells in T175 cm^2^ flask. Cells were co-transfected with 25 μg of pLuc/GFP transfer vector, 15 μg of p8.74 packaging vector, 13 μg of pMD2 pseudotyping vector, and 5 μg of pREV, diluted in 3 ml of complete Dulbecco’s modified Eagle’s medium (DMEM; Euroclone) without serum and 145 μl of PEI (Polysciences, Inc.; ratio 1:2.5 DNA-PEI). The transfection mixture was incubated for 15 min at room temperature, added with four times volume of complete DMEM without serum, and transferred to the cell monolayer.

After 6 h of incubation at 37°C with 5% CO_2_, the transfection medium was replaced with 25 ml of fresh complete medium and the cells were 48 h incubated at 37°C, 5% CO_2_. The flask was then frozen-thawed at –80°C; transfected cell supernatant (TCS) containing Luc/GFP lentivirus was clarified *via* centrifugation at 3,500 rpm for 5 min at 4°C, filtered through a 0.45-μm filter (Millipore), aliquoted, titered by limited dilution, and stored at –80°C.

### Creation of Stably Transduced Mouse Bone Marrow-Derived Mesenchymal Stem/Stromal Cells

Mouse bone marrow-derived mesenchymal stromal/stem cells clones #2 and #11 were seeded at a concentration of 10^5^ cells in a 25-cm^2^ flask with 5 ml of complete EMEM with 10% FBS for 4 h at 37°C in an atmosphere with 5% CO_2_. When the cells were attached, they were transduced with 5 ml of non-ultra-centrifuged Luc/GFP lentivirus. After 24 h, the medium was replaced with 5 ml of fresh complete medium, incubated at 37°C, and split when they reached confluence. The transduction efficiency of mBM-MSC and the percentage of GFP-positive/green fluorescent cells were evaluated by flow cytometry analysis using a FACS Canto II (BD Biosciences). Fluorescence intensity was determined with 50,000 cells per sample using a gated strategy for GFP signals based on the background signal from the non-transduced cells. Data acquisition and analysis were carried out with Diva 7 software (BD Bioscience). Luciferase expression was also assayed for the characterization of different transduced MSC clones before *in vivo* studies. *In vitro* titration of bioluminescence signals was obtained by diluting cells in PBS (1:2) starting from 10,000 cells/well. Then, 5 μl of luciferin per well was added, and photons emitted from transduced cells were immediately measured using IVIS (PerkinElmer Inc.) and quantified with the software Living Image^®^ version 4.3.1 (PerkinElmer Inc.). An average of photons/cells was reported.

### 3-[4,5-Dimethylthiazol-2-yl]-2,5 Diphenyl Tetrazolium Bromide Assay

Parental clone #2Luc/GFP and #2LucRi/GFP MSCs were seeded 1.5 × 10^4^ cells/well in 0.5 ml of complete medium in 48-multi-well plates. After 24, 48, 72, and 96 h from cell seeding, culture medium was replaced with 0.2 ml of 3-[4,5-dimethylthiazol-2-yl]-2,5 diphenyl tetrazolium bromide (MTT) powder (0.5 mg/ml thiazolyl blue tetrazolium bromide, Sigma) diluted in serum-free culture medium. Plates were then incubated for 4 h at 37°C. The deposition of violet crystals in the bottom well reveals the presence of metabolic active cells. The solution was then removed and crystals were dissolved in 0.2 ml of dimethyl sulfoxide (DMSO). Absorbance was immediately quantified using a plate reader (xMark Microplate Spectrophotometer, Bio-Rad) at 570 nm wavelength, and optical densities were quantified through the Microplate Manager Software 6 version 6.3 (Bio-Rad). At each time point of observation, the proliferation assay was measured in triplicate for both cell lines, and the assay was repeated at least twice with similar results.

### *In vivo* Studies

This study was conducted using female inbred C57BL/6 mice from ENV (San Pietro al Natisone, Udine, Italy) and CRL (Calco, Lecco, Italy) aged 7–8 weeks. Prior to use, mice were acclimatized for at least 5 days to the local vivarium conditions (20–24°C room temperature; 40–70% relative humidity; and 12 h of light–dark cycle), having free access to standard rodent chow and softened tap water. All animal experiments described herein were authorized by the official competent authority and approved by the intramural animal welfare committee for animal experimentation of Chiesi Farmaceutici under protocol number 733/2019-PR and comply with the European Directive 2010/63 UE, Italian D.Lgs 26/2014, and the revised “Guide for the Care and Use of Laboratory Animals.”

Parental clone #11Luc/GFP and #2LucRi/GFP MSC were firstly administered *via* IV or IT, 5 × 10^5^ cells/mouse and 10^6^ cells/mouse, respectively, in four different groups (*n* = 5) of C57BL/6 female mice (ENV). Subsequently, 10^6^ #2Luc/GFP cells/mouse were IT implanted in five C57BL/6 mice (ENV). After 14 days from instillation, mice were culled and lungs were isolated and homogenized. #2Luc/GFP cells were sorted by FACS and expanded. After 24-h incubation with growth medium supplemented with either FBS (Gibco; *n* = 5) or homologous serum (Sigma; *n* = 5), they were lung reimplanted (named #2LucRi/GFP) in C57BL/6 female mice (ENV) *via* IT (10^6^ cells/mouse).

Finally, a comparison between two different animal producers (ENV and CRL) was conducted: 10^6^ #2Luc/GFP cells cultured with FBS were IT administered in ENV (*n* = 5) and CRL (*n* = 5) C57BL/6 female mice. For *in vivo* and *ex vivo* bioluminescence detection, animals were slightly anesthetized with 2.5% isoflurane (IsoFlo, Zoetis) and shaved to allow longitudinal detection of *in vivo* bioluminescent imaging signal. On days 0, 7, 14, 21, and 28 from cell implantation, mice were IP injected with 200 μl/mouse luciferin substrate (15 mg/ml in saline, PerkinElmer Inc.) and slightly anesthetized with isoflurane (2.5%). After 15 min, they were imaged in supine position using IVIS. Bioluminescence from the chest of the mice was quantified in photons/s/cm^2^ using the software Living Image^®^ version 4.3.1 (PerkinElmer Inc.). At the endpoint experiment, after the *in vivo* imaging, mice were culled with an overdose of anesthetic followed by bleeding from the abdominal aorta, and lungs were harvested and imaged *ex vivo* using IVIS.

### Sorting of Bright Clone #2Luc/GFP Mouse Bone Marrow-Derived Mesenchymal Stromal/Stem Cells From Lung Homogenates

Mouse lungs (*n* = 8) were enzymatically digested at 37°C for 90 min with 0.4 mg/ml of collagenase P. Enzyme activity was neutralized with DMEM containing 10% FBS and centrifuged at 1,200 g for 10 min to obtain a cell suspension. Cells were then resuspended in PBS at a final concentration of 1 × 10^6^ cells/ml, and #2Luc/GFP mBM-MSCs were sorted by FACSAria III flow cytometer (BD Biosciences) equipped with two air-cooled lasers at 488- and 633-nm wavelengths. Data were analyzed by Diva software (BD Biosciences).

### Statistics

Statistical analyses were performed using Prism 8 software (GraphPad Software Inc., San Diego, CA, United States). Two-way analysis of variance was performed, followed by Sidak’s multiple-comparison *post hoc* test to compare different experimental groups. A *p* < 0.05 was considered significant.

## Data Availability Statement

The raw data supporting the conclusions of this article will be made available by the authors, without undue reservation.

## Ethics Statement

The animal study was reviewed and approved by all animal experiments described in the paper were authorized by the official competent authority and approved by the intramural animal-welfare committee for animal experimentation of Chiesi Farmaceutici under protocol number: 733/2019-PR and comply with the European Directive 2010/63 UE, Italian D.Lgs 26/2014 and the revised “Guide for the Care and Use of Laboratory Animals.”

## Author Contributions

GD, MD, and FS conceived the experiments. EF, VF, FM, LR, AM, GG, FS, and GD performed the experiments. GD wrote the manuscript. MD edited the final version. All authors contributed to the article and approved the submitted version.

## Conflict of Interest

EF, GV, and FS were employed by company Chiesi Farmaceutici S.p.A. The remaining authors declare that the research was conducted in the absence of any commercial or financial relationships that could be construed as a potential conflict of interest.
