# Supplementary material for: Persistency of Mesenchymal Stromal/Stem Cells in Lungs
Source: Front Cell Dev Biol. 2021 Jul 16;9:709225. doi: 10.3389/fcell.2021.709225 (PMC8322774; doi:10.3389/fcell.2021.709225)
Supplement: Supplementary file 1 [file Data_Sheet_1.docx]

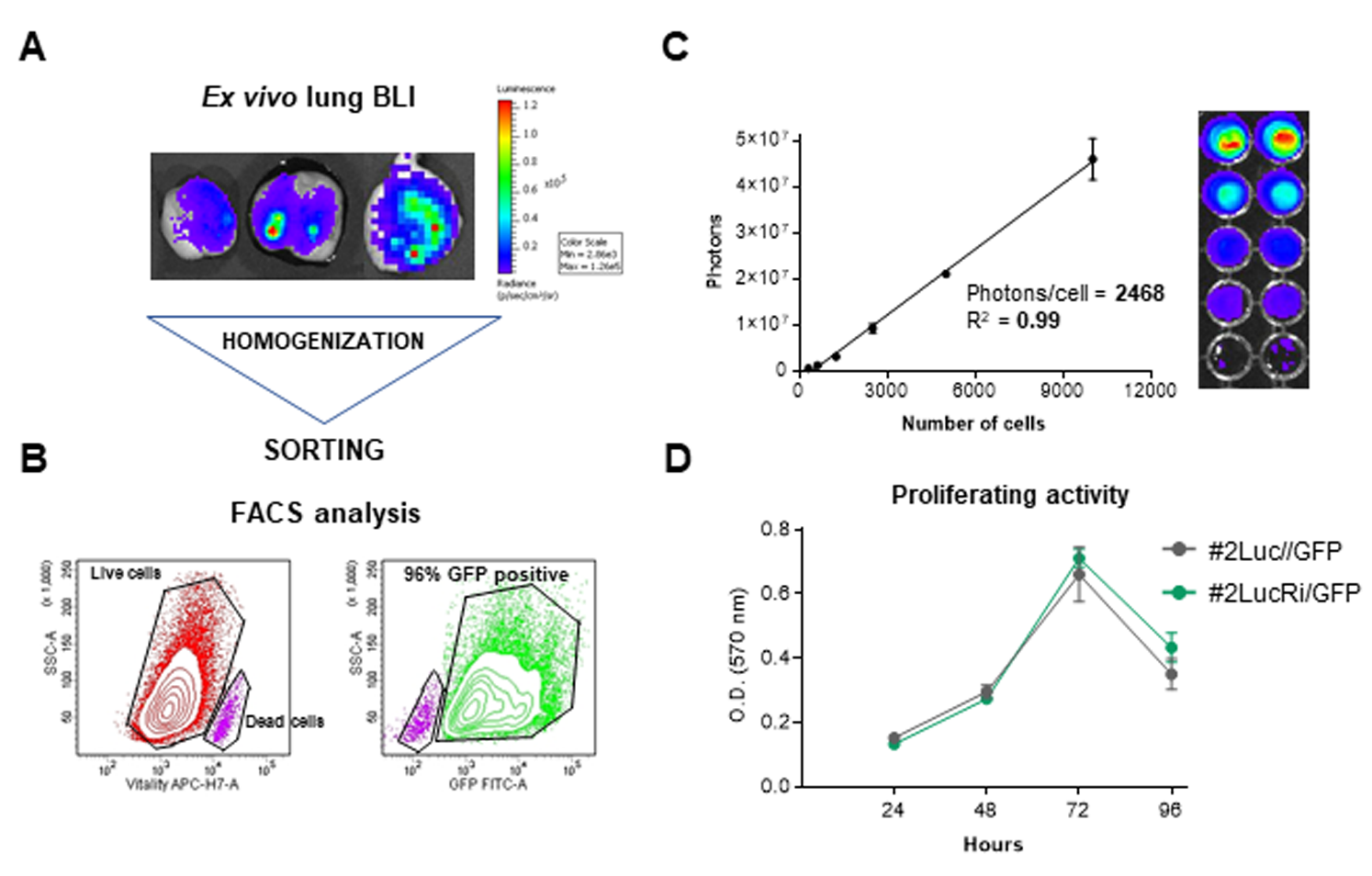


**Supplementary Figure 1.** Selection of mBM-MSCs clone #2LucRi/GFP. **A)** *Ex vivo* lungs BLI from ENV mice at 14 days after IT administration of mBM-MSC clone #2Luc/GFP (10^6^ cells/mouse). **B)** Cytofluorimetric analysis of pulled lung homogenates sorted on GFP fluorescence. **C)** *In vitro* BLI titration of clone #2LucRi/GFP cells. Total photons and number of plated cells were linearly correlated, and photons emitted per single cell were calculated. **D)** Cell viability assay (MTT) comparing #2Luc/GFP and #2LucRi/GFP cells (p>0.05; 2-way ANOVA followed by Sidak’s multiple comparison test).
